# The Many Faces of Urothelial Carcinomas: An Update From Pathology to Clinical Approach and Challenges in Practice

**DOI:** 10.5152/tud.2023.23023

**Published:** 2023-05-01

**Authors:** Duygu Enneli, Tolga Baglan

**Affiliations:** Department of Pathology, Ankara University School of Medicine, Ankara, Turkey

**Keywords:** Biomarkers, bladder cancer, fibroblast growth factor receptor, histological subtypes, immunotherapy, PD-L1 genomic testing, targeted therapies, urothelial cancer

## Abstract

Urothelial carcinoma is a heterogeneous disease with histomorphological and genomic variations throughout the same tumor or between tumors from different patients. It has been shown that most of these histologic and genetic differences have prognostic significance and may have a guiding role in determining the appropriate treatment choice for the patient. Therefore, it is crucial for both the pathologist and the clinician to be conscious of these variations and to consider them in patient management. Recently, a consensus molecular classification has been developed and categorized urothelial carcinomas into 6 subclasses. These molecular subclasses seem to be associated with prognosis and/or response to certain therapeutic approaches like chemotherapy or immune checkpoint inhibitory therapy; however, it has not yet been sufficiently validated and has some limitations for routine application. As is well known, there are therapeutic limitations in locally advanced or metastatic urothelial carcinomas, especially those inappropriate for standard therapy with platinum-based chemotherapy regimens. Emerging new therapeutic approaches and testing for appropriate patient selection for those are discussed in this article.

**Main Points**

- Histological subtypes of invasive urothelial carcinoma have prognostic and therapeutic significance. Therefore, it is important for both the pathologist and the clinician to be aware of these histological subtypes and to consider them in patient management.
- Urinary cytologic examination performed on urine samples is the most sensitive method for detecting high-grade urothelial neoplasms (high-grade urothelial carcinoma/urothelial carcinoma in situ) and should be combined with tissue-based evaluation on cystoscopic biopsy or transurethral resection.
- The appropriate PD-L1 immunohistochemical assay developed for the immune checkpoint inhibitory drug offered for the patient should be applied. If not possible, at least, matched evaluation method for that drug should be used to determine the PD-L1 status of the tumor.
- Sample from metastasis should be the first choice for the PD-L1 testing, if possible. If not, the patient’s last tumor block containing a sufficient amount of invasive urothelial carcinoma (at least 100 tumor cells) and with the least amount of necrosis or cautery artifact should be preferred.
- The risk stratification model of urothelial carcinomas should be improved by the integration of the gene expression profile of carcinomas. But the immunohistochemistry-based algorithm is needed for gene expression profiling, as it is a cheaper method to be implemented in clinical practice.

## Introduction

The most common histopathological type of bladder cancer is urothelial carcinoma (UC). Urothelial carcinoma is a costly tumor, that requires treatment for life, due to its high recurrence rate. Urothelium, from which UCs originate, has a high capacity for divergent differentiation and can give rise to a wide variety of morphological phenotypes or pathologies and frequently constitutes diagnostic pitfalls for pathologists with important implications on clinical management. Therefore, carcinomas of such a many-faced epithelium often present with various histopathological patterns/subtypes. Most of these histological subtypes (HS) are found to have prognostic importance and may guide treatment decisions.

The heterogeneous nature of UCs is now well known, and a better understanding of this tumor group and elucidation of novel molecular targets for new therapeutic options are the aims of many ongoing research. This review discusses some selected leading issues regarding UCs and aims to improve the clinicopathological correlation on handling and management of patients with UC. At first, morphological features and clinical importance of urothelial HSs are reviewed and then new reporting recommendations for urothelial proliferation that fall short of real papillary neoplasia are discussed, and the distinction of neoplastic/nonneoplastic lesions cannot be made clear. Molecular subclassification and its possible significance for clinical practice, emerging new therapeutic approaches, especially immune checkpoint inhibitory (ICI) therapy, and testing for appropriate patient selection are issues discussed further in the article.

### Histological Subtypes and Their Clinical Significance

Most of the bladder cancers (**~**75%) are pure conventional urothelial carcinomas (CUC), and a quarter contains HSs. These specific histological patterns often accompany areas of CUC at variable rates or may less frequently be pure. Different HSs may also coexist at varying rates in the same tumor. Many have significant prognostic or therapeutic implications for patients with UC.^1^ Recent reports have revealed that although each HS has its own characteristics, overall, UCs harboring any type of HS have a more aggressive course than pure CUCs.^1,2^ According to the current guidelines of the European Association of Urology (EAU) and Japanese Urology Association, UCs with any type of HS are considered “high risk,” even if they are not muscle invasive.^3,4^ Accurate diagnosis and reporting of HSs in the pathology report are critical in terms of risk stratification of patients, prediction of prognosis, and guiding treatment decisions.^5^

These subtypes have a diverse range of histological features that may imitate different types of nonurothelial malignancies. They may show varying degrees of atypia, from innocent-appearing tumor cells to anaplastic cells with marked atypia. Although histopathological criteria have been defined for all of these subtypes, the correct diagnosis of HSs harbors challenges in routine pathology practice, and consultation of a specialized genitourinary pathologist is often required. As there is no additional test to clarify the diagnosis of HSs, they are often overlooked and result in underreporting of UCs. This may lead to the failure in clinical management and progression of the disease, since patients who need aggressive treatment regimens may miss the chance of required therapy. A review of 589 UCs diagnosed in transurethral resection (TUR) specimens by genitourinary pathologists revealed that HSs have not been reported in 44% of UCs by general pathologists.^6^

There has not been definitive information on the true prevalence of these HSs, their impact on survival, and treatment options appropriate for them yet.^1,2,7,8^ Sampling limitations of tumors and high interobserver variability among pathologists understate the incidence of HSs.

Though recent studies have indicated the aggressive nature of most HSs, UCs with HSs (UC-HS) actually present at an advanced stage more frequently than pure CUCs; and survival outcomes do not show a significant difference when compared to pure CUCs of the same stage.^1^ Since the presence of ≥80% HS in a UC, regardless of the type, has been reported to be associated with tumor recurrence and mortality after radical cystectomy (RC), the pathology report should also include the percentage of HS.^9^ Radical cystectomy after neoadjuvant chemotherapy (NAC) and pelvic lymph node dissection are still the main treatment strategies for muscle-invasive UCs. However, HS-specific differences in treatment responses may require some changes in the treatment protocol, and some HS-specific molecular features may provide additional treatment options. Neoadjuvant chemotherapy has been found to be beneficial, especially in UCs with micropapillary, plasmacytoid, sarcomatoid and mixed subtypes, and neuroendocrine tumors.^10^

The HSs and their characteristic features are summarized in Table 1.

### Urothelial Carcinoma with Divergent Differentiation

#### Urothelial Carcinoma with Squamous or Glandular Differentiation

**Figure 1.**
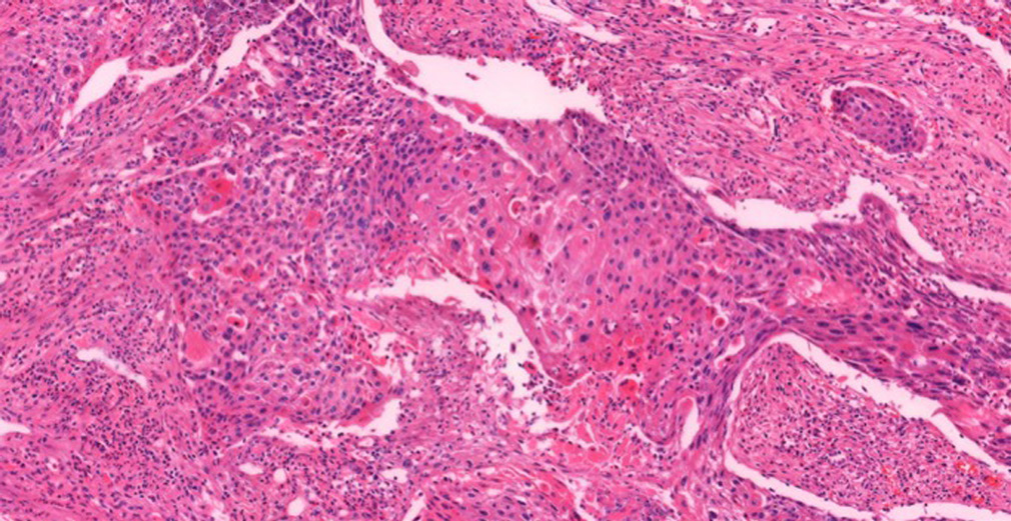
High-grade infiltrative urothelial carcinoma with squamous differentiation. Solid islands of polygonal cells and evidence of keratinization.

**Figure 2.**
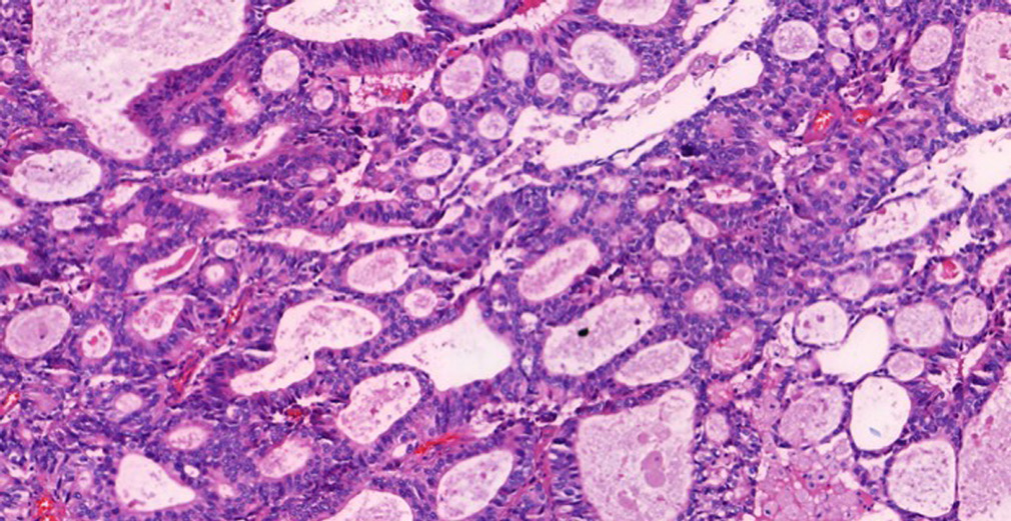
Glandular differentiation. True glandular spaces within the solid/trabecular areas of tumor cells.

Urothelial carcinoma with squamous differentiation (UC-SD) is the most common HS, with an incidence of 40% of infiltrative UCs. The presence of intercellular bridges and/or keratinization in the tumor indicates squamous differentiation^11^ (Figure 1). Glandular differentiation occurred in up to 18% of infiltrative UCs and was represented by the presence of intratumoral tubules or glands^12^ (Figure 2). Sometimes UCs with glandular differentiation (UC-GD) show extensive extracellular mucin, imitating colloid carcinoma, and can be characterized by cell nests (some with signet ring-like morphology) floating in extracellular mucin.

Tumors with any identifiable urothelial component—urothelial carcinoma in situ (U-CIS) or UC—in combination with areas of squamous or glandular differentiation are classified as “UC-SD” or “UC-GD,” respectively. If there is not any identifiable urothelial component and the tumor consists entirely of a squamous or glandular component, they are diagnosed as “squamous cell carcinoma (SCC)” or “adenocarcinoma,” respectively.

Although initial reports showed lower survival outcomes of UC-DD,^13^ it has been elucidated recently that UCs with squamous or glandular differentiation have a tendency to be diagnosed at a higher stage than pure UCs, but their survival does not differ from pure UCs at the same stage.^14^ The literature is conflicting in terms of the role of NAC in the treatment of these patients. Some studies state that the stage of tumor decreases after NAC, while others report poor response to NAC.^15^ The Southwest Oncology Group-directed Intergroup Study indicated that UC-SD/UC-GD responded better to NAC than pure UCs and showed better overall survival with NAC.^16^ Neoadjuvant chemotherapy and adjuvant chemotherapy (AC) seem to be beneficial in UC-SD and downsize the tumor mass in the urinary bladder. However, it may not be recommended for pure SCC, which is very rare.^17^ Urothelial carcinoma with squamous differentiation shows relatively higher expression of PD-L1, and ICI can be a therapeutic option for these patients.

#### Urothelial Carcinoma with Trophoblastic Differentiation

**Figure 3.**
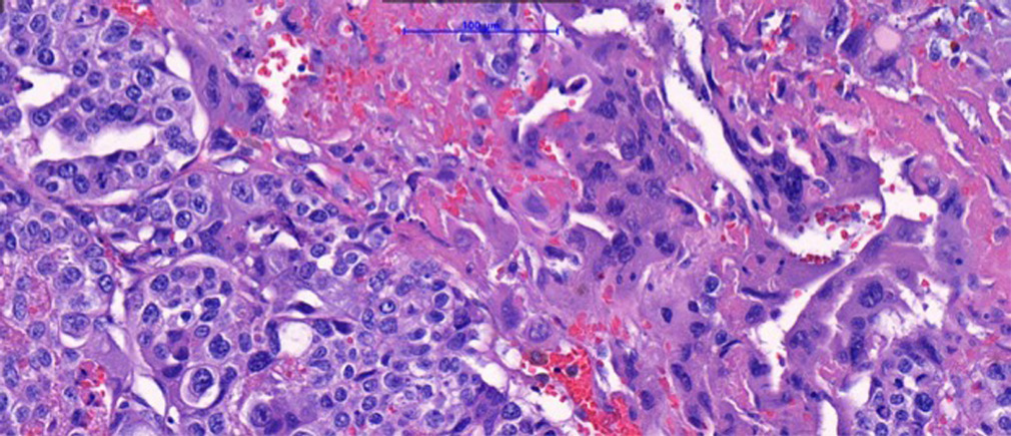
Trophoblastic differentiation. Large multinucleated syncytiotrophoblastic giant cells admixed with solid islands of mononucleated cells.

It is rare with an incidence of up to 5.5% of UCs of the bladder. It can be characterized by scattered aggregates of syncytiotrophoblasts (Figure 3) or areas of choriocarcinomatous differentiation in a background of CUC, or only β-HCG immunoexpression without any trophoblast.^18^ Studies suggest that UCs-TD tend to present at a higher stage, but the trophoblastic differentiation in UCs does not indicate poor outcomes in multivariate analysis.^19^

#### Nested Subtype Urothelial Carcinoma

**Figure 4.**
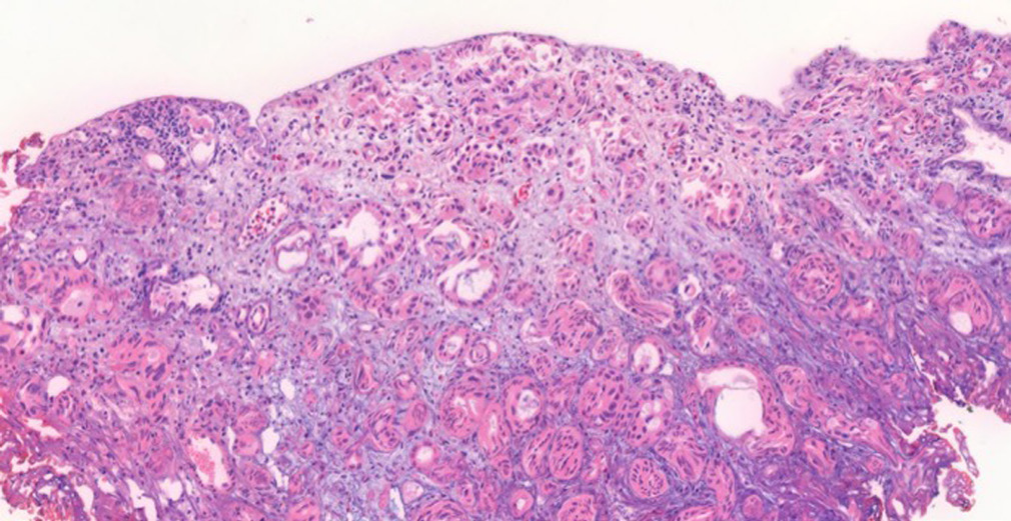
Nested urothelial carcinoma anastomosing, irregularly sized tumor cell nests with bland cytological features.

It is a rare subtype with a prevalence of 0.3% and is often underdiagnosed. It is characterized by small round to irregular nests of cells with bland cytological features (Figure 4). The most distinctive feature of this HS is its deceptively bland histological appearance, which may sometimes cause misdiagnosis of benign urothelial proliferative lesions, especially in biopsies, and the delay in correct diagnosis may lead to the presentation of nested subtype urothelial carcinoma (NV-UCs) at the advanced stage. Von Brunn nests, cystitis cystica, cystitis glandularis, paraganglioma, nephrogenic adenoma, and inverted papilloma are the leading entities in the differential diagnosis of NV-UC. Although some other histological features may help for the differential diagnosis, muscularis propria invasion is the most definitive histological finding in favor of carcinoma, since benign mimics are not observed in the muscularis propria. And the atypia of tumor cells may become more prominent in the deeply invading parts of the tumor. An immunohistochemical examination does not provide much benefit, but since the majority of NV-UCs harbor TERT promoter mutations, detection of these mutations by molecular examination may be useful in distinguishing the nested subtype of UC from its benign mimics.^20^ In case of the presence of firmly packed urothelial cell nests in the bladder mucosa with any grade of cytological atypia, especially in a biopsy specimen, extreme care should be taken not to miss the diagnosis of NV-UC.

Despite of deceptively bland histological appearance, NV-UCs behave like high-grade CUCs of the same stage. The NV-UCs have a higher tendency of muscle invasion, extravesical disease, and metastases. In a study of 52 patients with UC, the rate of locally advanced disease in RC specimens was higher in NV-UCs than in pure UCs. However, when stages of both tumor groups were matched, there was not a significant difference between study groups at a median follow-up of approximately 11 years, in terms of relapse-free (RFS) or cancer-specific survival (CSS).^21^

#### Large Nested Subtype Urothelial Carcinoma

Large nested subtype urothelial carcinoma (LNV-UC) was under the category of NV-UC in the previous 2016 World Health Organization (WHO) classification, but it has recently been considered a different HS in the 2022 WHO book due to its distinctive morphological and molecular aspects.^22^

The large nested subtype of UC is characterized by larger, well-circumscribed, or confluent tumor cell nests, again with innocent cytological features. As LNV-UC frequently harbors *FGFR3* mutations, *FGFR3* inhibitors may be a therapeutic choice.^23^

#### Microcystic and Tubular Urothelial Carcinoma

**Figure 5.**
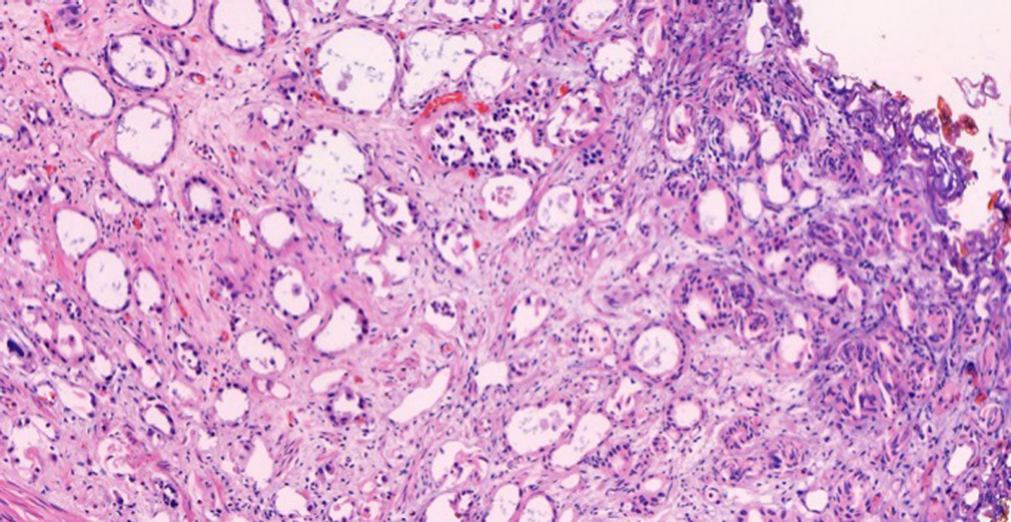
Microcystic and tubular urothelial carcinoma. Scattered tubular structures or microcysts, lined by a single layer of cuboidal or flattened tumor cells with bland cytology.

Microcystic and tubular urothelial carcinoma (MCV-UC) is characterized by cysts or tubular structures in varying sizes (1-2 mm), lined by cuboidal/depressed epithelial cells, often with intraluminal secretions (Figure 5). Tumor cells have deceptively bland cytology similar to the NV-UC. This subtype usually presents at an advanced stage with muscularis propria invasion but shows similar survival outcomes compared with pure CUC at the same stage, like NV-UC, though reports for this HS are very limited.^24^

#### Micropapillary Urothelial Carcinoma

**Figure 6.**
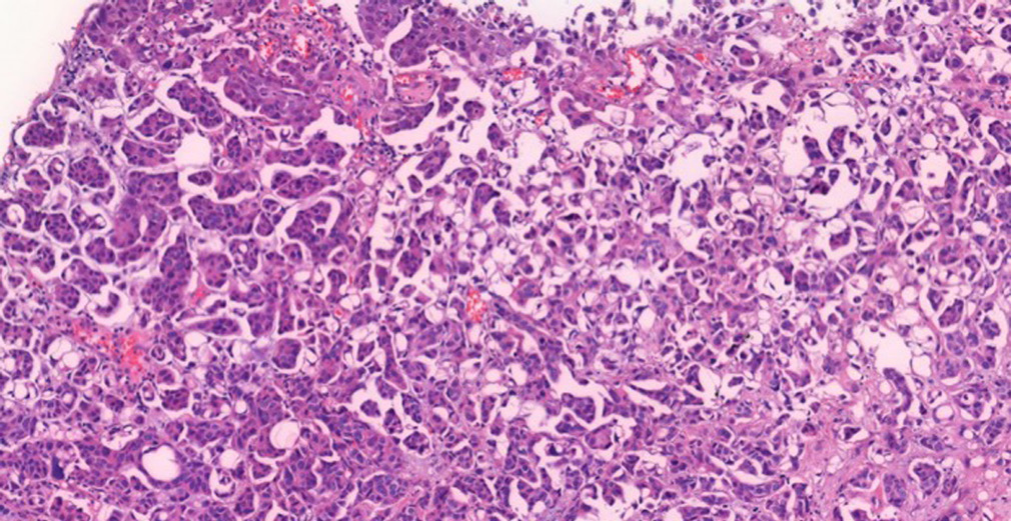
Micropapillary urothelial carcinoma. Small nests of tumor cells with surrounding lacunar space and without fibrovascular cores. In some areas, there are multiple tumor nests within a lacunae.

Micropapillary urothelial carcinoma (MPV-UC) is an aggressive HS, comprising 2%-5% of all UCs. It shows small tumor cell nests located in lacunar/retraction spaces (Figure 6). These retraction spaces can often mimic vascular invasion, and the absence of endothelial cell marker expression by immunohistochemistry (IHC) may help to exclude the lymphovascular invasion.

Interobserver reproducibility for the decision of micropapillary pattern is moderate (kappa = 0.54), with 93% agreement among experienced uropathologists. The presence of more than one tumor nest within the same lacuna and tumor rings are the most characteristic features for the diagnosis of MPV-UC.^25^ The micropapillary pattern can also be seen on the surface of noninvasive UC, and this should not be considered as MPV-UC. The WHO classification underlines this issue and recommends that micropapillary pattern on the surface of non-invasive UC should not be reported by the pathologist to prevent misunderstanding as its more aggressive invasive counterpart and hence protect the patient from unnecessary treatment.

It is more prone to lymphovascular invasion, U-CIS, and lymph node involvement and is more often diagnosed at an advanced stage.^26^ Some studies have reported that MPV-UC has a worse prognosis than conventional UC, and its prognostic effect has been found to correlate positively with the proportion of micropapillary components.^27^ Willis et al^28^ detected a significant difference in terms of 5-year CSS between stage cT1 MPV-UCs who underwent early RC and those who received Bacillus Calmette-Guérin (BCG) immunotherapy (100% vs. 60%, respectively, *P* = .006), and it has been found that the presence of the >25% micropapillary component in a UC is related to more frequent disease progression in the course of BCG therapy^28^; therefore, it has been suggested that cT1 UCs with a very small focus on micropapillary pattern may get benefit from BCG therapy.^29^

The EAU—European Society for Medical Oncology (ESMO) consensus recommends RC, even in cases of stage T1 MPV-UCs.^30^ But, it has recently been reported that MPV-UC did not show worse RFS, CSS, or overall survival (OS) than conventional UC after RC, when adjusted for the clinicopathologic properties such as tumor stage, surgical margin status, and administration of chemotherapy.^31^ The rate of upstaging of T1 MPV-UC in early RC is high (ranging from 23% to 73%).^32^ There are conflicting data on the efficacy of NAC in the treatment of MPV-UC. Neoadjuvant chemotherapy administration has been found to provide significant downstaging in RC, although it may not be as effective on survival as in CUCs.^31,33^

Micropapillary urothelial carcinoma harbors ERBB2 gene changes (amplification and mutation) more frequently than CUC and other UC-HSs. Therefore, the paraffin block determined for the HER2 testing of UCs with mixed histology must necessarily contain the micropapillary component of the tumor.

#### Sarcomatoid Urothelial Carcinoma

Sarcomatoid urothelial carcinoma** (**S-UC) is rare with an estimated prevalence of 0.1%-0.3%.^34^ Risk factors such as radiation and intravesical cyclophosphamide therapy are shown to predispose to the development of this aggressive subtype.

Its morphological diversity makes the diagnosis problematic. It can imitate several mesenchymal neoplasms of the urinary bladder. Diagnosis of S-UC requires at least one of the following criteria: The detection of epithelial origin should be demonstrated morphologically or by cytokeratin expression immunohistochemically. Another criterion is the detection of immunohistochemical GATA3 expression especially in the sarcomatous component.^35^ Recent molecular data indicated that sarcomatoid differentiation is an expected end for all types of epithelial bladder tumors.^36^ Sarcomatoid urothelial carcinoma is a biphasic malignant tumor with both epithelial and mesenchymal components, identified morphologically and/or immunohistochemically. These components most probably originate from a common malignant clone. The epithelial component can be CUC, SCC, adenocarcinoma, or high-grade neuroendocrine carcinoma and is frequently admixed.

**Figure 7.**
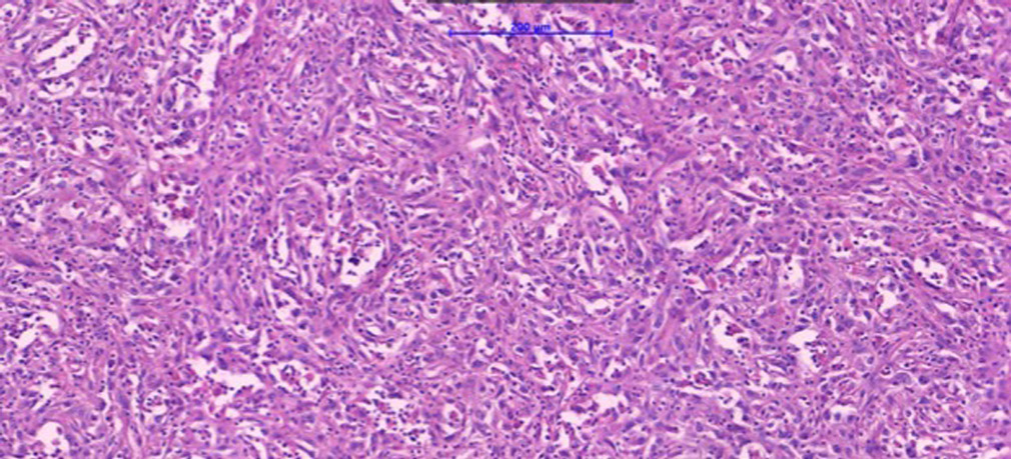
Sarcomatoid urothelial carcinoma. Sarcomatoid areas with elongated spindle cells and high-grade cytological atypia.

The sarcomatoid histology is frequently an undifferentiated high-grade sarcoma with spindle cells, round cells, or pleomorphic giant cells (Figure 7). Accompanying heterologous sarcomatous components have been found to be related to decreased survival (osteosarcoma, chondrosarcoma, rhabdomyosarcoma, etc.), and if present, should be mentioned in the pathology report.^36^

A large study of 489 S-UCs revealed that patients who underwent RC alone and those who received NAC or AC had similar survival outcomes.^37^ The poor response of sarcomatoid UC to chemotherapy has been confirmed by some other studies.^38^ Contrary to these, it has also been reported that NAC provides a survival advantage in these patients.^39^ Sarcomatoid urothelial carcinoma presents at an advanced stage more frequently than CUC and is associated with worse survival.^38^

As it is a rare histological subtype, little is known about it. Multicenter studies with larger series are needed to determine whether it has an independent prognostic role and which treatment approach is more effective. As they show PD-L1 overexpression, anti-PD-1/PD-L1 therapy can be a treatment choice for these patients.^40^

#### Plasmacytoid Urothelial Carcinoma

**Figure 8.**
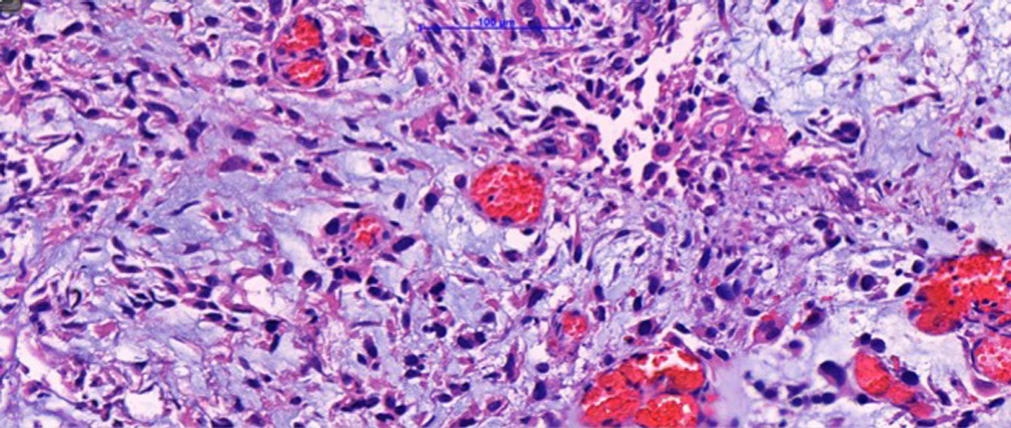
Plasmacytoid urothelial carcinoma. Discohesive infiltrating tumor cells with eccentrically placed nuclei and abundant eosinophilic cytoplasm.

**Figure 9.**
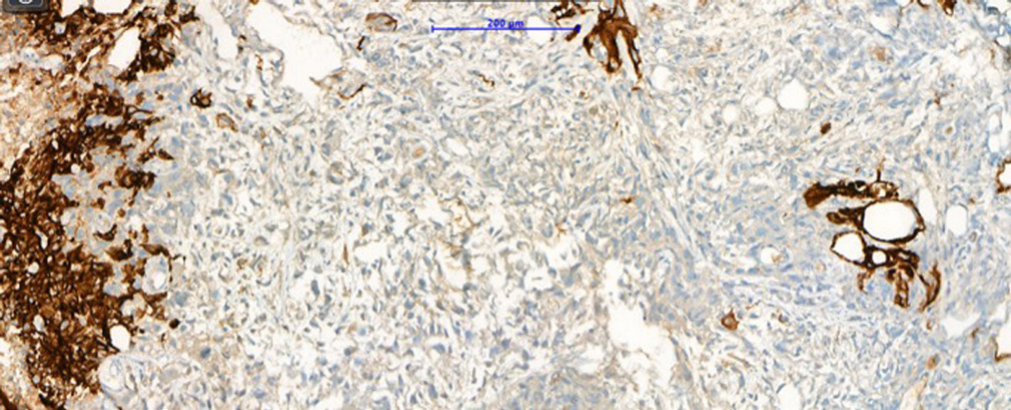
E-cadherin immunohistochemical staining. Aberrant loss of E-cadherin membranous expression in the tumor cells with plasmacytoid morphology. Note intact membranous staining in overlying urothelium.

Discohesive tumor cells histologically resembling plasma cells are the characteristic cells of plasmacytoid urothelial carcinoma **(**PV-UC) (Figure 8). Signet ring-like cells containing intracytoplasmic vacuoles are also frequently seen.^2^ Gastric signet ring cell carcinoma, breast lobular carcinoma, and plasma cell myeloma are the leading entities in the differential diagnosis of this HS. Immunohistochemistry helps in the differential diagnosis in most of the cases. Most of them (84%) exhibit E-cadherin loss immunohistochemically, resulting from the truncating mutations of the encoding gene CDH1 or, rarely, the CDH1 promoter hypermethylation. Plasmacytoid urothelial carcinoma (PV-UC) can be diagnosed usually by its characteristic morphology, but the immunohistochemical evaluation of E-cadherin expression in tumor cells can be applied for confirmation (Figure 9). Differential diagnosis with some entities may require immunohistochemical examination.

Molecular changes in the CDH1 gene may be responsible for the marked discohesion and increased invasiveness of tumor cells, and thus its frequent advanced stage at presentation.^41^ Peritoneal carcinomatosis is common.^42^

This HS is not an independent prognostic factor, with similar prognosis and survival outcomes to pure CUC at the same stage. The EAU-ESMO consensus does not recommend RC for pT1 cases.^30^ Marked discohesion of tumor cells and the lack of desmoplastic stromal reaction around them make difficult to determine the tumour-normal tissue boundary during the surgery and this results in a higher rate of positive surgical margins. Tumor cell infiltration can be seen away from the macroscopically visible disease. Therefore, preoperative diagnosis of PV-UC is critical, as it requires wider excision to obtain intact surgical margins during RC.^42,43^ The high recurrence rate of this HS is thought to be a result of frequent surgical margin positivity.

Treatment efficacy in PV-UC is uncertain. Plasmacytoid urothelial carcinoma has a poor response to chemotherapy, resulting in poor survival even in patients whose tumor stage is downstaged to pT0 by RC.^44^ Despite NAC, most are unresectable for surgery. Urgent novel treatment options other than standard cisplatin-based chemotherapy are needed for the treatment of this HS.^45^ Plasmacytoid urothelial carcinoma also does not appear to be a suitable candidate for ICI therapy due to frequently low PD-1/PD-L1 expression rates^46^

### Other Very Rare Histological Subtypes

#### Lymphoepithelioma-Like Urothelial Carcinoma

It is more common in males between the fifth and seventh decades and, like other HSs, is often associated with a UC component.

This HS consists of syncytial sheets/nests of large pleomorphic cells, obscured by dense mixed-type inflammation and resembles morphologically to nasopharyngeal lymphoepithelioma-like carcinoma but differs in that it is not related to Epstein–Barr virus (EBV) or human papilloma virus (HPV) infection.^47^

In its pure form, lymphoepithelioma-like urothelial carcinoma (LL-UC) responds well to platinum-based chemotherapy and does not show a high potential for metastasis.^48^ But it frequently coexists with CUC or other HSs, and the accompanying tumor types mainly determine the prognosis. They frequently show PD-L1 overexpression (93%),^49^ and ICI therapy may be a treatment option for this HS.

#### Giant Cell Urothelial Carcinoma

This HS is characterized by bizarre, pleomorphic, multinucleated tumor giant cells, frequent necrosis, and atypical mitosis. It is a very aggressive tumor that presents frequently at advanced stage.^50^ Differentiating these bizarre cells from trophoblastic or osteoclast-like giant cells of other HSs can be challenging. In case of the tumor consisting entirely of giant cells, secondary carcinomas infiltrating the bladder, especially giant cell carcinoma of the lung, should be excluded. Epithelial and urothelial marker expression, immunohistochemically, in at least some of the tumor giant cells may be helpful in diagnosis. The detection of concomitant CUC or U-CIS, albeit focal, is very helpful in favor of the diagnosis of giant cell UC.

#### Clear Cell (Glycogen-Rich) Urothelial Carcinoma

Clear cell (glycogen-rich) urothelial carcinoma (CC-UC) is composed of tumor cells with abundant clear cytoplasm due to the accumulation of intracytoplasmic glycogen. It should be differentiated from primary bladder clear cell adenocarcinoma or metastatic clear cell carcinoma from different sites (kidney, prostate, female genital tract) or perivascular epithelioid cell tumor (PEComa) originating from the bladder. CK7, p63, and GATA3 positivity of the tumor cells indicate urothelial origin. It should also be considered in the differential diagnosis that the thermal effect may cause cytoplasmic clearing in tumor cells in TUR samples.

In most cases, there is also an accompanying CUC which is a diagnostic histological finding in favor of CC-UC.

As this HS is very rare, little is known about prognosis/treatment modalities. However, it seems to have a poor prognosis, as frequently diagnosed at advanced stage.^51^

#### Lipid-Rich Urothelial Carcinoma

Lipid-rich urothelial carcinoma (LR-UC) is also an uncommon HS characterized by lipoblast-like cells containing one or more intracytoplasmic vacuoles with lipid content, indenting the nucleus. This HS should be differentiated from UC-GD with signet ring cells, heterologous liposarcoma component of S-UC, and liposarcoma. It is usually associated with CUC or other HSs. Poor 5-year survival outcome (5-year survival—42%) was reported in a large multi-institutional study.^52^

#### Poorly Differentiated Urothelial Carcinoma

Poorly differentiated urothelial carcinoma (PD-UC) is a very rare entity that has not been clarified yet. It lacks morphological features of urothelial origin but has evidence of urothelial lineage by IHC. This HS is often seen as an undifferentiated carcinoma rich in osteoclastic giant cells, with histologic resemblance to the giant cell tumor of bone representative morphology of this subtype. Immunohistochemistry is required to demonstrate epithelial differentiation in this tumor. The accompanying CUC or U-CIS component is diagnostic for PD-UC. Although data are very limited, it is more likely to be an aggressive tumor.

#### “Intermediate” Proliferative Urothelial Lesions Without Clear Cytological Atypia and Fall Short of Papillary Neoplasia

Urothelial proliferations, which were previously named as “urothelial hyperplasia/papillary urothelial hyperplasia,” have recently been found to be associated with low-grade papillary urothelial carcinoma (LG-PUC), indicating that these proliferative lesions are more likely precursors of LG-PUC. Therefore, in the previous 2016 WHO classification, the terminology "urothelial proliferation with uncertain malignant potential" was recommended for these lesions. However, as this terminology is ambiguous, it has no longer been included in the last WHO classification (5th edition), and no distinct terminology has also been recommended for these lesions. These are considered as “Early low-grade noninvasive papillary carcinoma” or as an extension of such a lesion.^53^ On the other hand, the Genitourinary Pathology Society proposes the term “atypical urothelial proliferation (AUP)” for such hyperplastic lesions. They are frequently detected on follow-up cystoscopies of patients with a previous diagnosis of papillary urothelial neoplasia.

**Figure 10.**
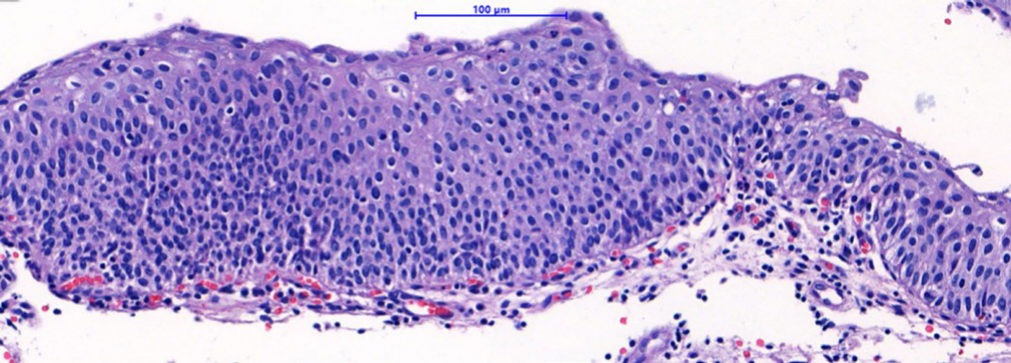
Atypical urothelial proliferation, flat. Flat, thickened, proliferative urothelium with minimal nuclear atypia.

**Figure 11.**
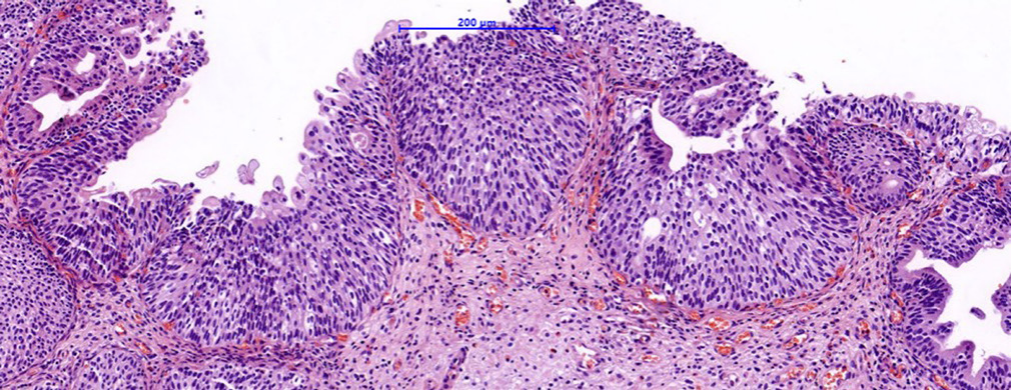
Atypical urothelial proliferation, tented. Slight ondulations of thickened, proliferative urothelium without any true papillary structure with a fibrovascular core. It falls short of real papillary neoplasia. There is not any true papilla formation, branching, or detached papillary fronds. Higher magnification of the same lesion is illustrated in Figure 12

**Figure 12.**
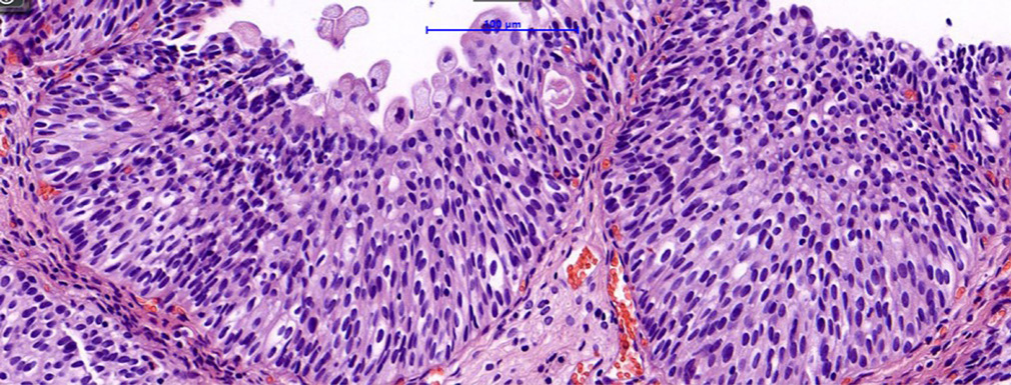
Atypical urothelial proliferation, tented. Minimal cytological atypia.

They are characterized by thickened urothelial epithelium without nuclear atypia or with minimal atypia, as seen in reactive conditions. The thickened proliferative epithelium can be flat or shows slight/moderate ondulations without any true papillary structure with a fibrovascular core and falls short of real papillary neoplasia. Recommended terminologies are “AUP-flat (formerly called as flat urothelial hyperplasia)” (Figure 10) and “AUP-tented (formerly called as papillary urothelial hyperplasia)” (Figures 11 and 12), respectively. Any true papilla formation, branching, or detached papillary fronds contradict this definition and are diagnosed as papillary urothelial neoplasia.^54^ At cystoscopy, AUP-tented is frequently defined as a focal lesion with variable appearances (papillary/raised/sessile, etc.).

They often represent a transition lesion to synchronous papillary urothelial neoplasm and very rarely occur in a de novo setting (without a synchronous or prior diagnosis of papillary urothelial neoplasia). When they occur in a de novo setting, AUP-tented seems to be more related to the development of urothelial neoplasia than AUP-flat. The AUP-tented is probably a precursor lesion of LG-PUC, as these patients have an increased risk of having LG-PUC, previously, synchronously, or subsequently.^54^ In order to elucidate the clinical significance of de-novo AUP lesions, comprehensive studies are needed. When the diagnosis of AUP-flat or AUP-tented is given, a comment should also be added to the report: "AUP may be precursor lesion of low-grade papillary urothelial neoplasms or may represent a shoulder lesion of a concurrent papillary urothelial neoplasm. This should be considered for the patient management plan and clinical follow-up of the patient is recommended.”

#### Urinary Cytology: An Easily Accessible and Valuable Diagnostic Tool for Urothelial Carcinoma Screening That Should Not Be Neglected

Cytologic examination of urine or bladder washing sample is a sensitive method to detect high-grade urothelial neoplasms (high-grade UC/U-CIS) and should be applied in conjunction with cystoscopic biopsy or TUR, since discohesive highly atypical neoplastic cells can frequently be shed from the mucosal surface into the urine. Especially if the epithelium is denuded and not clearly visible in the cystoscopic biopsy, urine cytological examination should definitely be applied as a complementary screening method for high-grade urothelial neoplasms (UC or U-CIS).

The Paris system, a standardized, universally accepted reporting method in urine cytology since 2015, should be used for reporting.^55^ It has high specificity (97.8%) and sensitivity (ranges from 83.3 to 87.1%) on recognition of high-risk urothelial neoplasms.^56^ In case the cystoscopy is negative and the urine cytology is positive, a closer follow-up of the patient with repeating biopsies and examining the upper urinary tract are critical.

### Molecular Subclassification of Urothelial Carcinoma and Its Possible Significance for Clinical Practice

In recent years, the personalized medicine approach has been increasingly preferred, especially for the treatment of patients with advanced carcinoma and this leads to an increasing need for molecular subtyping of carcinomas. Molecular profiling provides prognostic information and guides the determination of the appropriate treatment regimen specifically for that patient. Breast cancer is a good model in terms of the clinical success of molecular classification. The molecular subclasses of breast carcinomas can be successfully determined immunohistochemically with a limited number of antibodies, and this brings high clinical applicability at a low cost.

Several molecular classifications based on gene expression profiles have also been developed for UCs since 2014.^57,58^ Then these concepts were unified under a consensus molecular classification, which proposed 6 molecular subclasses for muscle-invasive bladder cancers: luminal papillary (LumP) (24%), luminal nonspecified (LumNS) (8%), luminal unstable (LumU) (15%), stroma-rich (15%), basal/squamous (35%), and neuroendocrine-like (NE-like) (3%) UCs.^59^ However, molecular classification of UCs has not been implemented in routine application yet, due to several limitations. Since UCs show histological diversity even in the same tumour, they are also genetically heterogeneous tumors , and therefore, it is difficult to classify the tumor into a particular molecular category. The efficacy of molecular classification for UCs in clinical practice has not been validated prospectively yet, and the high cost of molecular testing is another big obstacle. Due to financial reasons and the need for prospective evidence for its efficacy, the EAU-ESMO consensus does not require molecular classification in selecting appropriate patients for treatment.

Molecular subtyping provides prognostic information for patients and has a predictive role in treatment response. Certain HSs are found to be associated with specific molecular subtypes (Table 1). In addition, various antibody panels have been proposed to perform molecular subtyping by IHC, which is a much lower-cost test.^58,60,61^

Luminal unstable, basal/squamous, and NE-like subgroups are found to be associated with poor survival, whereas LumP, LumNS, and stroma-rich tumors have better survival.

Basal/squamous and NE-like subtypes appear to be suitable candidates for neoadjuvant therapy with platinum-based chemotherapy^59,62-64^ as NAC prolongs CSS and OS in these patients.

Basal/squamous tumors show high epidermal growth factor receptor expression and respond well to treatments targeting this pathway.

Luminal papillary subtype is found to be rich in FGFR3 gene abnormalities and responds well to Erdafitinib, a tyrosine kinase inhibitor.^65^

### Emerging New Therapeutic Approaches for Advanced Urothelial Carcinoma

#### Immune Checkpoint Inhibitory Therapy

#### Sample Selection for PD-L1 Testing

There is no definite recommendation regarding sample selection for the PD-L1 test (Table 3). It can be performed on TUR material, cystectomy, lymph node, or metastatic tissue. In order to come through intratumoral heterogeneity problems, the test can be done in more than one sample (e.g., 2 or more blocks of the same TUR) in some cases, but a high agreement has been reported between small biopsy specimens and cystectomy specimens.^70^ Intratumoral heterogeneity may also be associated with HS, if present (squamous differentiation areas in the tumor tend to show higher PD-L1 expression, whereas plasmacytoid differentiation tends to show lower, and the block selection should be done accordingly).^71^

The use of biopsies of metastatic sites, if present, should be preferred, since PD-L1 results differ between primary UC and its metastasis.^72^ It is not recommended on decalcified specimens or smear samples, and specimens achieved shortly after chemotherapy or immunotherapy (e.g., cystectomy specimens of patients undertaken NAC) should not be used for PD-L1 testing too. The selected tissue block for the test should contain an invasive component at least, as it is expected to represent the advanced-stage tumor better. If there is not any sample containing the infiltrative component of the tumor, a new biopsy (ideally from a metastatic site) with invasive UC should be requested. When more than one tissue sample with invasive UC is available, the last tumor block containing adequate infiltrative component (at least 100 TCs) and the tumor block with the least amount of necrosis or cautery artifact should be preferred.

Adequate fixation in 10% neutral buffered formalin for 12-24 hours (1/5 to 1/10 tissue to formalin ratio) is important for the reliability of the test. Overfixation (>72 hours) or underfixation should be avoided as these reduce immunoreactivity. A positive control should be present on the same slide, and tonsil tissue with sufficient amount of lymphoid tissue should be preferred as a positive control.

Some studies on various cancer types have revealed that digital image analysis increases the accuracy of PD-L1 testing; therefore, if there is an opportunity for a digital analysis system, it should be preferred for the evaluation of PD-L1 expression.^73^

#### Fibroblast Growth Factor Receptor Inhibition

The *Fibroblast Growth Factor Receptor (FGFR)* is a family of tyrosine kinase receptors consisting of 4 transmembrane receptors, FGFR1-4. It stimulates cellular proliferation and angiogenesis by induction of the PI3K-AKT, PKC, and Ras/MAPK paths. Abnormal *FGFR* signaling directly contributes to tumor development by stimulating cancer cell growth and angiogenesis.^74^ A wide variety of cancers harbor *FGFR* aberrations with UCs being the most common at a rate of 32%^75^ and the most common *FGFR* aberrations in UCs are mutations (18.3%); followed by copy number amplifications (8.7%) and translocations (7.1%).^75^ Detection of genetic alterations involving the FGFR3 gene (mutations or gene fusions) provides guidance to predict response to FGFR inhibitors.

Erdafitinib, which is an *FGFR (FGFR1-4)* tyrosine kinase inhibitor, has recently been accepted by the FDA for locally advanced or metastatic, platinum-resistant UCs harboring *FGFR2/3* mutation or fusion. The response rate to this drug has been reported as about 40% and increases up to 59% after the administration of immunotherapy.^65^ Four point mutations (p.R248C, p.S249C, p.G370C, and p.Y373C) and 2 fusions (TACC3v1 and TACC3v3) in the *FGFR3* gene, detected in RNA samples of carcinoma tissues, provide prediction in terms of the responsiveness to *FGFR* inhibitors. There are some other aberrations of *FGFR2* and *FGFR3* genes (*FGFR3*:*BAIAP2L1, FGFR2:BICC1,* and* FGFR2:CASP7*) in UCs, which are not considered in determining appropriate patients for erdafitinib therapy, as the clinical utility of them have not been demonstrated yet. Particularly, LumP molecular subtype of UC is rich in *FGFR3* gene abnormalities and has high responsiveness to erdafitinib.^76^

As we know, platinum-based chemotherapy comes first in NAC and AC in UC and in the treatment of metastatic disease. Low response rates to platinum-based systemic chemotherapy have been reported for UCs with *FGFR3* aberrations. According to the study by Teo et al, none of the patients with *FGFR3* aberrations had a complete treatment response or shorter RFS after platinum-based NAC.^77^ *FGFR3* gene changes in UCs have also been related to poorer response to ICI therapy, possibly due to the immune-deprived phenotype, through stimulation of the *PI3K/AKT* pathway.^65^

#### Antibody-Drug Conjugates

Antibody-drug conjugate (ADC) is a novel, promising therapeutic approach which consists of a target-specific monoclonal antibody and a cytotoxic anticancer agent covalently bound to each other via a linker. The antibody targets specific antigens expressed on the tumor surface, providing the cytotoxic agent delivered selectively to TCs.^78^ enfortumab vedotin is an ADC, consisting of an antibody against nectin 4, which is a cell adhesion molecule highly expressed in UC cells and conjugated antineoplastic payload [mono-methyl auristatin E (MMAE)]. Nectin-4 antibody mediates the antineoplastic agent to specifically target TCs.

Locally advanced or metastatic UCs, especially those who previously received platinum-based chemotherapy and ICI, are considered incurable due to the lack of a successful treatment option for this patient group. Treatment with enfortumab vedotin provided a 44% objective response rate (12% complete response rate) and a 7.6-month duration of response in those patients.^79^ Based on these findings, the FDA has accepted the use of enfortumab vedotin for patients who have previously received a combination of platinum-based chemotherapy and immunotherapy with a PD-1/PD-L1 inhibitor, or for cisplatin inappropriate patients who have been administered one or more therapy choices.

#### Her-2 Testing

*HER2* testing is implemented in routine practice for gastric/breast cancers, with established guidelines. But the importance or prevalence of *HER2* expression in UCs has not been clarified yet, and *HER2* testing in UCs is not applied in routine clinical practice.

*HER2* testing for UCs is applied in 2 situations. One is to investigate *HER2* gene amplification to determine the probability of getting benefits from *HER2*-targeted therapies such as trastuzumab and tyrosine kinase inhibitors (apatinib, neratinib, and lapatinib). The therapeutic value of these agents for breast and gastric carcinomas is clear, whereas they do not seem to be so effective in UCs.^80^ The second and more recent indication is to investigate any level of *HER2* expression in TCs, to determine the eligibility of patients for novel treatments, recently developed to target *HER2*+ UC cells, such as ADCs.^80,81^ For example, disitamab vedotin (RC48) is an ADC composed of an anti-HER2 antibody bound to a cytotoxic agent with antineoplastic activity. The use of RC48 has recently been accepted by FDA for *HER2*-positive, locally advanced/metastatic UCs, in which prior platinum-based chemotherapy has failed.^82,83^

**Figure 17.**
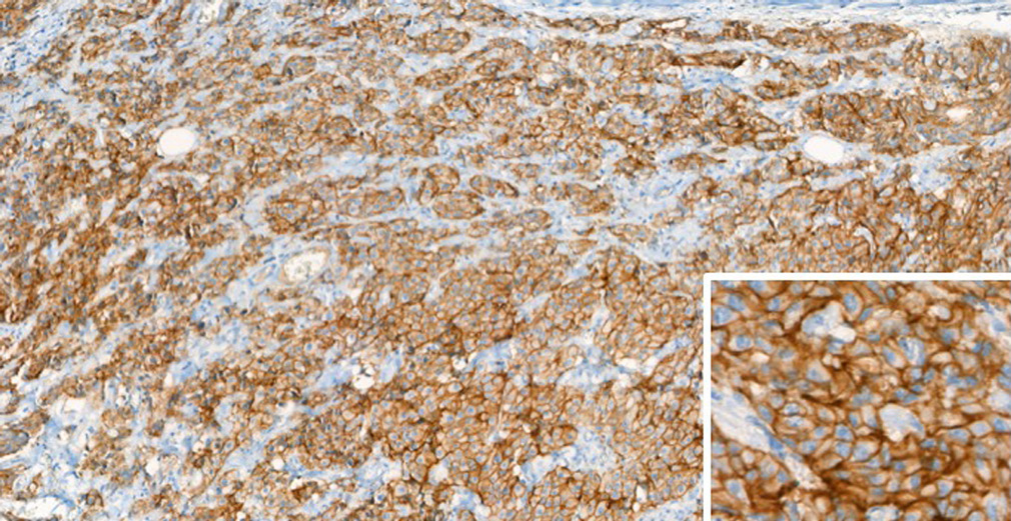
Complete and strong membranous HER2 immunostaining in tumor cells of urothelial carcinoma, exceeding 10% (3 +).

There is a great need to develop a standardized HER2 test to determine patients who may get benefit from *HER2*-targeted therapies in UCs. Studies in the literature show variability regarding the definition of the *HER2*(+) status in UCs. American Society of Clinical Oncology (ASCO) / the College of American Pathologists (CAP) scoring guidelines developed for breast and gastric carcinomas are widely used for UCs. Accordingly, an IHC score of 3+ is described as HER2(+) status (Figure 17). On the other hand, carcinomas that are +2 positive with IHC and show *HER2* gene amplification by in situ hybridization (ISH)+/ fluorescence in situ hybridization (FISH) method are also considered *HER2* (+). The administration of anti-*HER2* therapies, such as trastuzumab, requires *HER2*(+) status, whereas carcinoma cells with any level of HER2 expression may be a potential target for *HER2-*targeted ADCs. Therefore one more category—“*HER2*-low” is reported in the literature and described as an IHC score of 2+ and a negative ISH/FISH or IHC score of 1+.^84^ And *HER2*-negative status is described as the absence of any membranous expression in TCs. Therefore, the interpretation of *HER2* expression varies according to the treatment agent to be used.

## Conclusion

It seems that significant progress has been achieved in the clinical approach to UCs, but there still seems to be a long way to go, especially for locally advanced or metastatic UCs. Patients should be managed with close communication between the pathologist and the clinician by being aware of all the data we currently have and using them in the most appropriate way. Current risk stratification models of patients with UC are based on clinical and histopathological parameters, and since molecular differences seem to be the main factor leading to clinical variations, there is a need for a sufficiently validated gene expression profiling model in which molecular features are also integrated. But mRNA-based profiling models are too expensive to apply for routine practice, and immunohistochemistry-based models should definitely be emphasized.

## Figures and Tables

**Figure 13.**
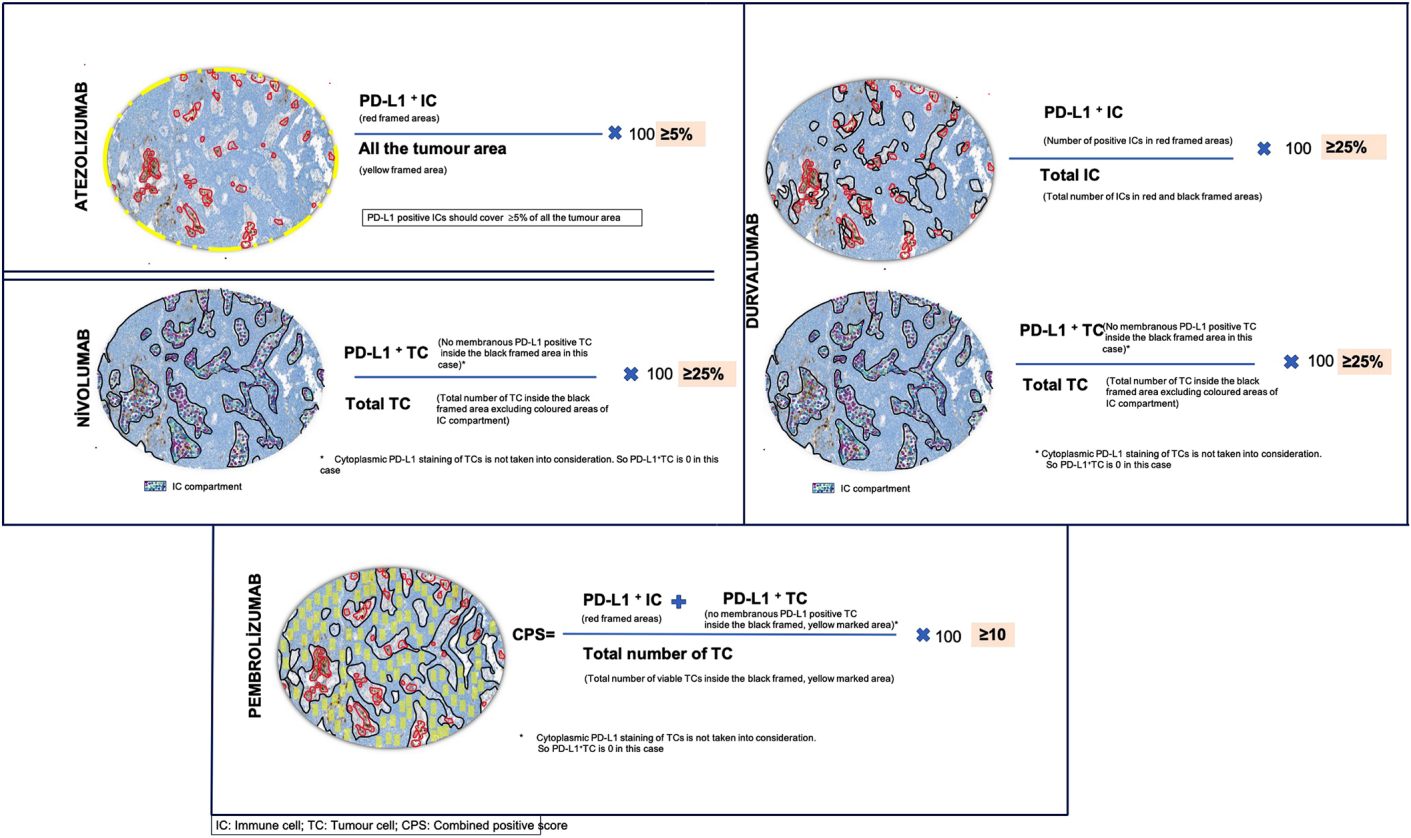
Illustrations of PD-L1 scoring algorithms applied in urothelial carcinoma. PD-L1 testing in conjunction with atezolizumab (VENTANA SP142): Patients are eligible for atezolizumab treatment if PD-L1 positive ICs cover ≥5% of all the tumor area. PD-L1 testing in conjunction with nivolumab (PharmDx 28-8): Patients are eligible for nivolumab treatment if the proportion of TCs with PD-L1 staining to total TCs is ≥25%. PD-L1 testing in conjunction with durvalumab (VENTANA SP263): Patients are eligible for durvalumab treatment if the proportion of ICs with PD-L1 staining within the total IC area or the proportion of TCs with PD-L1 staining within the total TC area is ≥25%. PD-L1 testing in conjunction with pembrolizumab (PharmDx 22C3): Patients are eligible for pembrolizumab treatment if CPS (the proportion of TCs and ICs with PD-L1 staining to total TC area) is ≥25%. CPS, combined positive score; IC, immune cell; TC, tumor cell.

**Figure 14.**
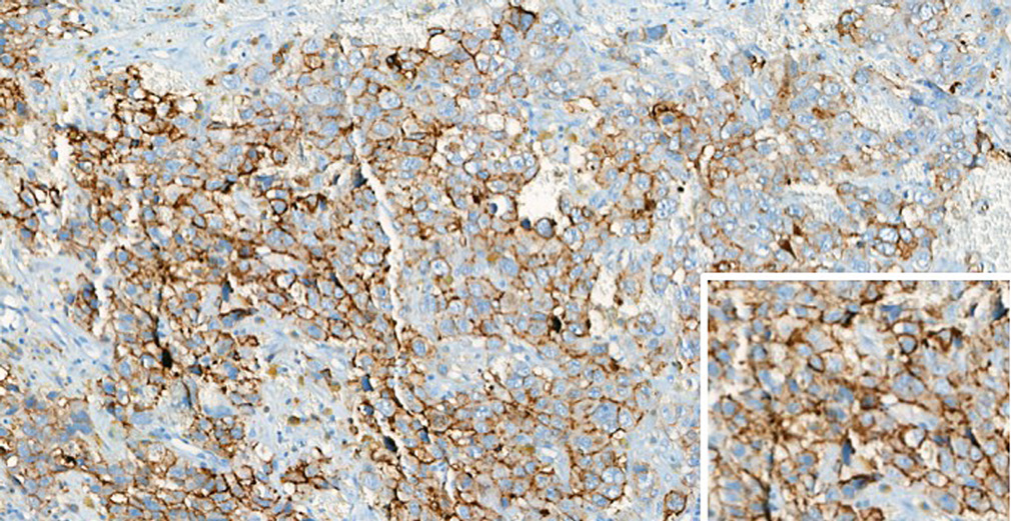
High PD-L1 expression in urothelial carcinoma. Membranous PD-L1 immunostaining in tumor cells exceeding 25%.

**Figure 15.**
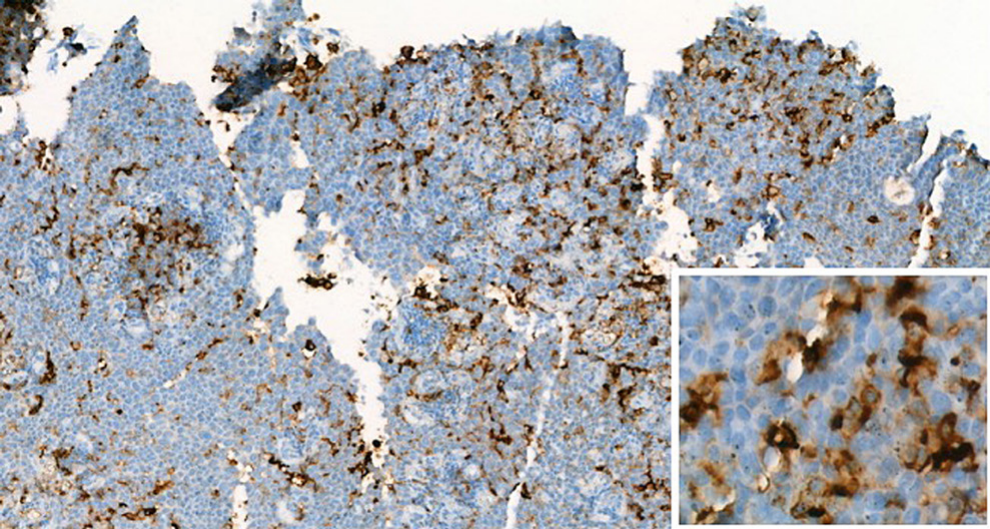
Cytoplasmic, granular immunostaining of tumor cells, which is not considered, in the evaluation of PD-L1 status. Only membranous staining is considered positive in tumor cells.

**Figure 16.**
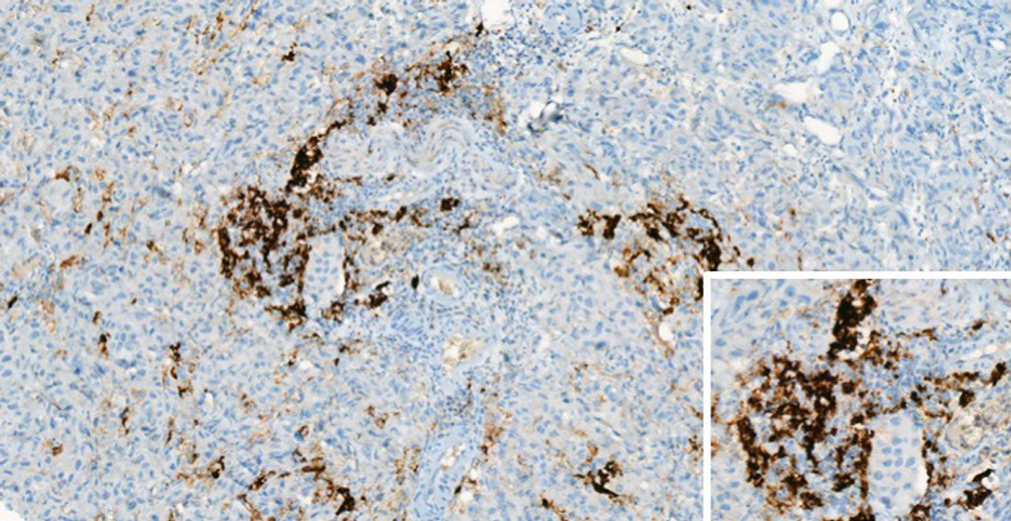
High PD-L1 expression in urothelial carcinoma. PD-L1 immunostaining (any type of staining—cytoplasmic or membranous) in immune cells exceeding 25%.

**Table 1.** Histological Subtypes and Their Characteristic Features Are Summarized

Subtype Histology	Incidence	Histological Features	Molecular Subclass	Therapeutic Choices	Overall Survival (OS)
Squamous differentiation	40%	The presence of intercellular bridges and/or keratinization in the tumor	Basal/squamous	NAC, RC, PLND, ACT	Higher stage at presentation than pure UC
				**High PD-L1 expression**	Similar OS to pure UC with the same stage
Glandular differentiation	18%	The presence of intratumoral tubules or glands	Luminal, unstable	NAC, RC, PLND, ACT	Higher stage at presentation than pure UC
		Rarely, cell nests floating in extracellular mucin mimicking colloid-like carcinoma (some with signet ring cell morphology)				Similar OS to pure UC with the same stage
					Trophoblastic differentiation	5.5%	Scattered aggregates of syncytiotrophoblasts or areas of choriocarcinomatous differentiation in a background of CUC, or only β-HCG immunoexpression but no recognizable trophoblasts	Unknown		Higher stage at presentation than pure UC
Similar OS to pure UC with the same stage									
Nested subtype	0.3%	Small round to oval irregular urothelial cell nests with bland cytology	Luminal unstable/basal/squamous	NAC, RC, PLND	Higher stage at presentation than pure UC
					Similar OS to pure UC with the same stage
Large nested subtype		Larger irregular and infiltrating urothelial cell nests with bland cytology	Luminal papillary	NAC, RC, PLND	Higher stage at presentation than pure UC
				***FGFR3 *** **mutations ­ (Anti-** ***FGFR3 *** **therapy is an option)**	Similar OS to pure UC with the same stage
Microcystic and tubular subtype		Round to oval cysts of varying sizes (1-2 mm) or tubular structures lined by cuboidal/flattened urothelial cells, often containing intraluminal secretions and have a deceptively bland cytology	Luminal unstable/Basal/squamous	NAC, RC, PLND	Higher stage at presentation than pure UC
					Similar OS to pure UC with the same stage
Micropapillary subtype	2-5%	Small nests of tumor cells in lacunar/retraction spaces	Luminal, NOS	-Poor response rate to intravesical BCG -Early RC may improve survival Many patients upstaged at RC -Efficacy of NAC is controversial -***ERBB2 (HER2*** **) amplification is frequent (** ***ERBB2*** ** targeted therapy may be an option)**	More likely to present in the advanced stage than CUC
		The presence of multiple tumor nests within the same lacunar space and tumor rings are the most characteristic features				Controversial whether this HS is an independent poor prognostic factor? OR	Is reduced survival related to advanced stage at presentation?
					Plasmacytoid subtype	~1-3%	Discohesive cells histologically resembling plasma cells. Signet ring-like cells containing intracytoplasmic vacuoles also frequent	Luminal	RC (role of NAC before cystectomy has been questioned. Poor response to cisplatin)	More likely to present in advanced stage than CUC
										Controversial whether this HS is an independent poor prognostic factor? OR
Need for treatment options	Is reduced survival related to advanced stage at presentation?							
Sarcomatoid subtype	0.1-0.3%	Morphology is highly variable	Basal	NAC, RC, PLND	More likely to present in advanced stage than CUC
		Frequently an undifferentiated high-grade spindle cell sarcoma				Controversial whether this HS is an independent poor prognostic factor? OR	Is reduced survival related to advanced stage at presentation?
					Lymphoepithelioma-like		Syncytial sheets, nests, and cords of undifferentiated cells with large pleomorphic nuclei and prominent nucleoli.	Basal/squamous	Combination therapy	
							A dense infiltration of mixed inflammatory cells obscuring the epithelial cells is a characteristic feature of this tumor.		including RC The impact of NAC is undetermined	
may also consist of round cells or pleomorphic giant cells and may contain heterologous components such as osteosarcoma, chondrosarcoma, or rhabdomyosarcoma.	**High PD-L1 expression**									
	Giant cell		Bizarre, pleomorphic, multinucleated tumor giant cells. Necrosis and atypical mitotic figures are easily noticed.	Unknown			**-**		**-**	
Clear cell		Sheets of polygonal cells with abundant clear cytoplasm	Unknown	**-**	**-**
Lipid rich		Lipoblast-like cells containing one or more intracytoplasmic vacuoles indenting the nucleus	Luminal	**-**	**-**
Poorly differentiated UC		Lack morphological features of urothelial origin, but have evidence of urothelial lineage by immunohistochemistry	Unknown	**-**	**-**
		Undifferentiated carcinoma with osteoclastic giant cells is the most frequent histology			

ACT, adjuvant chemotherapy; BCG, bacillus Calmette-Guérin; CUC, conventional urothelial carcinoma; HS, histological subtype; NAC, neoadjuvant chemotherapy; OS, overall survival; PLND, pelvic lymph node dissection; RC, radical cystectomy; UC, urothelial carcinoma.

**Table 2.** Details of Administration of Immune Checkpoint Inhibitor Agents for Patients with Advanced Urothelial Carcinoma

Approved ICI Agents	Approved PD-L1 Assay	Evaluated Component	Cut-off for PD-L1 Positivitiy	Details	US, FDA-Indication	Europe, EMA-Indication
Atezolizumab	VENTANA PD-L1 (SP142)	IC	5% (PD-L1 stained IC covering ³5% of the tumor area)	All ICs are included except plasma cells	Locally advanced or metastatic UCs	Locally advanced or metastatic UCs
					a. Cisplatin ineligible and PD-L1 positive (detected by an FDA approved PD-L1 assay)	b. Cisplatin ineligible and PD-L1 positive (detected by Ventana SP142 PD-L1 assay)
					OR	OR
					c. Ineligible for platinum-based therapy or progressed during or after (within 12 months) platinum-based therapy, regardless of PD-L1 status	d. Previously received platinum-based chemotherapy
Durvalumab	VENTANA PD-L1 (SP263)	IC, TC	25% (Both IC and TC)	All ICs are included except plasma cells	Locally advanced or metastatic UCs, progressed during or after (within 12 months) platinum-based chemotherapy	
Pembrolizumab	PD-L1 IHC 22C3 pharmDx	IC, TC	CPS³ 10	PD-L1 stained slide must have at least 100 viable TCs	Locally advanced or metastatic UCs	Locally advanced or metastatic UCs
					Cisplatin ineligible and PD-L1 positive (CPS ³ 10)	Cisplatin ineligible and PD-L1 positive (CPS ³ 10)
			CPS=[PD-L1 positive cells (TC, IC) / Total number of viable TCs × 100]	Only staining in lymphocytes and macrophages is considered as IC+	OR	OR
					Ineligible for platinum-based therapy or progressed during or after (within 12 months) platinum-based chemotherapy Regardless of PD-L1 status	Previously received platinum-based chemotherapy
Nivolumab	PD-L1 IHC 28–8 pharmDx	TC	5%		Locally advanced or metastatic UCs, progressed during or after (within 12 months) platinum-based chemotherapy	

CPS, combined positive score; IC, immune cell; ICI, immune check point inhibitory; TC, tumor cell; UC, urothelial carcinoma.

**Table 3.** Key Points About the PD-L1 Testing

The appropriate PD-L1 immunohistochemical assay developed for the ICI drug that will be offered should be applied. If not possible, at least matched evaluation method for that ICI drug should be used to determine the PD-L1 status of the tumor. Sample from metastasis should be the first choice for the PD-L1 test. If not, the patient’s last tumor block should be preferred to contain a sufficient amount of invasive UC (at least 100 tumor cells) and with the least amount of necrosis or cautery artifact. If no tissue sample with invasive component is available, the pathologist should request a new biopsy (ideally from a metastatic site) containing invasive UC. If there is any HS in the tumor, the selected tumor block for PD-L1 test should contain the component that has high propensity for high PD-L1 expression (e.g., squamous differentiation). Decalcified specimens or smear samples and specimens obtained immediately after chemotherapy or immunotherapy (e.g., cystectomy specimens of patients undertaken NAC) are not suitable for the PD-L1 test. Tumor sample does not require any special preparation for the PD-L1 test, other than adequate fixation (in 10% neutral buffered formalin for 12-24 hours, 1/5 to 1/10 tissue to formalin ratio). Fixation for 24 hours at room temperature is considered optimal, and overfixation (>72 hours) or underfixation should be avoided as these reduce immunoreactivity. Positive control should be present on the same slide, and tonsil containing sufficient lymphoid tissue should be the first choice as positive control. If there is an opportunity for digital analysis system, it should be used for the evaluation of PD-L1 expression.
